# Interference in DNA Replication Can Cause Mitotic Chromosomal Breakage Unassociated with Double-Strand Breaks

**DOI:** 10.1371/journal.pone.0060043

**Published:** 2013-04-03

**Authors:** Mari Fujita, Hiroyuki Sasanuma, Kimiyo N. Yamamoto, Hiroshi Harada, Aya Kurosawa, Noritaka Adachi, Masato Omura, Masahiro Hiraoka, Shunichi Takeda, Kouji Hirota

**Affiliations:** 1 Department of Radiation Genetics, Graduate School of Medicine, Kyoto University, Yoshidakonoe, Sakyo-ku, Kyoto, Japan; 2 Group of Radiation and Tumor Biology, Career-Path Promotion Unit for Young Life Scientists, Kyoto University, Yoshidakonoe, Sakyo-ku, Kyoto, Japan; 3 International Graduate School of Arts and Sciences, Yokohama City University, Yokohama, Japan; 4 Radiation Oncology and Image-Applied Therapy, Kyoto University Graduate School of Medicine, Kyoto, Japan; University Medical Center Hamburg-Eppendorf, Germany

## Abstract

Morphological analysis of mitotic chromosomes is used to detect mutagenic chemical compounds and to estimate the dose of ionizing radiation to be administered. It has long been believed that chromosomal breaks are always associated with double-strand breaks (DSBs). We here provide compelling evidence against this canonical theory. We employed a genetic approach using two cell lines, chicken DT40 and human Nalm-6. We measured the number of chromosomal breaks induced by three replication-blocking agents (aphidicolin, 5-fluorouracil, and hydroxyurea) in DSB-repair-proficient *wild-type* cells and cells deficient in both homologous recombination and nonhomologous end-joining (the two major DSB-repair pathways). Exposure of cells to the three replication-blocking agents for at least two cell cycles resulted in comparable numbers of chromosomal breaks for *RAD54^−/−/^KU70^−/−^* DT40 clones and *wild-type* cells. Likewise, the numbers of chromosomal breaks induced in *RAD54^−/−/^LIG4^−/−^* Nalm-6 clones and *wild-type* cells were also comparable. These data indicate that the replication-blocking agents can cause chromosomal breaks unassociated with DSBs. In contrast with DSB-repair-deficient cells, chicken DT40 cells deficient in PIF1 or ATRIP, which molecules contribute to the completion of DNA replication, displayed higher numbers of mitotic chromosomal breaks induced by aphidicolin than did *wild-type* cells, suggesting that single-strand gaps left unreplicated may result in mitotic chromosomal breaks.

## Introduction

Morphological analysis of chromosomal aberrations in mitotic cells is widely used for the diagnosis of leukemia and the identification of mutagenic chemical agents [1], [2]. Chromosomal aberrations include chromosomal breakage, fusion, and translocation [3]. According to the International System for Human Cytogenetic Nomenclature (ISCN), chromosomal breakage, i.e. the discontinuity of sister chromatids, is classified into two types: chromatid-type breaks, which involve discontinuity in one of the sister chromatids, and isochromatid-type breaks, which involve discontinuity in both sister chromatids at the same location [4] (Figure S1). Chromosomal breaks are induced by a variety of mutagenic agents, such as ionizing radiation [5]–[8]. It is generally believed that virtually all chromosomal breaks are associated with DSBs at the site of the break. This idea is supported by experimental data. DSBs introduced by restriction endonucleases indeed induce chromosomal breakage, as well as translocation [9]–[13]. Additionally, chromosomal breaks and subsequent chromosomal translocation are frequently observed at genes encoding antigenic receptors in lymphocytes derived from patients with Ataxia Telangiectasia Mutated (ATM) dysfunction and lymphocytes deficient in DSB repair [8], [14]–[17].

Chromosomal breaks are caused not only by DSB-inducing agents such as ionizing radiation, but by chemical agents that repress DNA replication [18]. Such agents include aphidicolin, 5-fluorouracil (5-FU), and hydroxyurea (HU). Aphidicolin is a reversible inhibitor of replicative DNA polymerases [19], [20]. 5-FU, when metabolized to fluorodeoxyurideine, is a potent inhibitor of thymidylate synthase, and thereby depletes TTP pools and promotes dUTP incorporation into chromosomal DNA [21]. HU reduces dNTP levels by inhibiting the ribonucleotide reductase enzyme [22]. Although these drugs, as well as ionizing radiation, are capable of inducing chromosomal breaks, it has not previously been determined whether or not they induce chromosomal breaks by generating DSBs.

DSBs are repaired by two major pathways: homologous recombination (HR) and nonhomologous end-joining (NHEJ) [23], [24]. The RAD54 protein significantly promotes HR-mediated DSB repair [7], [25], [26], while the KU70/KU80 proteins and ligase IV (LIG4) are all essential for NHEJ [27]. HR and NHEJ play a substantially overlapping role in DSB repair, as evidenced by the fact that cells deficient in both RAD54 and KU70 are considerably more sensitive to ionizing radiation than are cells deficient in either RAD54 or KU70 [7], [28], [29]. Accordingly, DSB-inducing chemical agents can be identified by detecting reduced cell viability and an increase in the frequency of chromosomal breakage in a DSB-repair-deficient mutant, compared to *wild-type* cells [30].

We here employ a genetic approach to analyze the cause of mitotic chromosomal breaks induced by three replication-blocking agents: aphidicolin, 5-fluorouracil, and hydroxyurea. We compared the number of induced chromosomal breaks in *wild-type* cells and in cells deficient in both HR and NHEJ. Interestingly, the agents induced comparable numbers of chromosomal breaks in both human Nalm6 and chicken DT40 cell lines [31], [32], indicating that interference with DNA replication can cause mitotic chromosomal breakage that does not result from DSB. To gain an insight into the nature of aphidicolin-induced mitotic chromosomal breaks, we analyzed chicken DT40 cells deficient in PIF1 or ATRIP. PIF1 facilitates DNA-replication-fork progression when forks slow down and encounter barriers on template strands [33]–[35]. ATR kinase also contributes to the completion of DNA replication by preventing replication-fork collapse when replication forks are stalled. The absence of PIF1 or ATRIP causes marked increases in the number of aphidicolin-induced mitotic chromosomal breaks. The data suggest that single-strand gaps due to incomplete DNA replication may represent mitotic chromosomal breaks.

## Materials and Methods

### Cell Lines and Culture Conditions

DT40 cell line is derived from chicken B lymphoma [32] and was cultured in RPMI 1640 medium (Nacalai Tesque, Kyoto, Japan) supplemented with 10% heat-inactivated fetal bovine serum (FBS) (AusgeneX, QLD 4210, Australia), 1% chicken serum (GIBCO-BRL, Grand Island, NY, USA), 50 µM mercaptoethanol (Invitrogen), L-glutamin (Nacalai Tesque), 50 U/mL penicillin, and 50 µg/mL streptomycin (Nacalai Tesque). The cell lines were maintained at 39.5°C in a humidified atmosphere and 5% CO_2_. Isogenic DNA-repair-deficient cell lines with disruption of two DNA repair genes (*RAD54/KU70* genes for HR and NHEJ) or either gene were used.

Nalm-6 cell line is derived from human pre-B cell [31] and was cultured in RPMI 1640 medium (Nacalai Tesque) supplemented with 10% heat-inactivated FBS, 50 µM mercaptoethanol (Invitrogen), L-glutamin (Nacalai Tesque), 50 U/mL penicillin, and 50 µg/mL streptomycin (Nacalai Tesque) at 37°C in a humidified atmosphere and 10% CO_2_. We used *wild-type* Nalm-6 cells and their isogenic mutant, the *RAD54^−/−/^LIG4^−/−^* clone, which is deficient in the two major DSB repair pathways (*RAD54* gene for HR and *LIG4* gene for NHEJ). Nalm-6 has a stable near-diploid karyotype, though it carries a reciprocal translocation between chromosome 5 and chromosome 12, t(5;12)(q33.2;p13.2) [36].

### Disruption of *PIF1* in DT40 Cells

*PIF1* disruption constructs were generated from genomic PCR products combined with *neo^R^* and *bsr^R^* selection-marker cassettes. Genomic DNA sequences were amplified using primers 5′- GGTCGACATCAAGAACAATTTCTTCTCATAAAGAGTG-3′ and 5′- GGACTAGTACACCACAACTTGATTCAACAACACTGAAA-3′, and the amplified 4.2 kb fragment was cloned into the *Sal*I-*Spe*I sites of a pBlueScript SK vector. Marker-gene cassettes, *neo^R^* and *bsr^R^* selection-marker genes, flanked by loxP sequences, were inserted into the *Bam*HI site of the amplified fragment to generate *PIF1*-*neo^R^* and *PIF1-bsr^R^*. To generate *PIF1^−/−^* cells, *wild-type* DT40 cells were transfected sequentially with *PIF1-neo^R^* and *PIF1-bsr^R^*. The 0.5 kb PCR fragment from genomic DNA, using primers 5′- TTCCCGAACCTCCTCATCACTTTACAGT-3′ and 5′- CTGCACAGCCAGTGAAGAAGACACTCTT-3′, was used as a probe for Southern blot analysis to screen gene-targeting events. Targeting efficiency for the first and second allele was 78% (18/23) and 42% (5/12), respectively.

### Chromosomal Aberration Analysis

For chromosomal aberration analysis of the DT40 cells, we prepared chromosome samples as previously reported [5]. The chicken karyotype consists of 80 chromosomes: 11 major autosomal macrochromosomes, the 2 ZW sex chromosomes, and 67 microchromosomes [37]. Giemsa-stained metaphase cells were scored at 1000× magnification, with scoring limited to the 11 major macrochromosomes and the Z chromosome [37]. Chromosomal aberrations (CAs) were classified as isochromatid or chromatid gaps, breaks, and exchanges (fusions including triradial, quadriradial, ring, dicentric, or other) according to the ISCN system [4]. We used a different protocol for chromosomal aberration analysis of the Nalm-6 cells. Briefly, compound-treated *wild-type* and DNA-repair-deficient clones were incubated at 37°C for 48 h. To arrest cells in the metaphase, 0.06% colcemid (GIBCO-BRL) was added 2 h before harvest. Cells were pelleted by centrifugation, resuspended in 5 mL of 0.6% sodium citrate for 20 min at room temperature, and fixed in 2 mL of a freshly prepared 3∶1 mixture of methanol:acetic acid (i.e., Carnoy’s solution). The pelleted cells were then resuspended in 1 mL of Carnoy’s solution and dropped onto clean glass slides and air dried. The slides were stained with a 5% HARLECO Giemsa stain solution (Nacalai Tesque) for 10 min, rinsed with water, and dried. All chromosomes in each mitotic cell were scored at 1000× magnification.

### Flow-cytometric Analysis to Measure Cell Viability

Annexin V (BioVision, Mountain View, CA, USA) assays were performed after treatment with drugs or irradiation with γ-ray. Flow-cytometric analysis was performed with a FACS Calibur flow cytometer (Beckton Dickinson, Mountain View, CA). Data were acquired and analyzed using CellQuest software.

### Cell Counting and Cell-cycle Analysis

Cell numbers were determined by mixing a PI-stained sample with a fixed number of 25 μ microspheres (Polysciences Inc., Warrington, PA), which can be distinguished from cells by forward and side-scatter characteristics during flow-cytometric analysis. Beads and living cells were counted simultaneously as gated events, and cell numbers were calculated. We analyzed the growth curve of each clone at least three times.

For cell-cycle analysis, cells were labeled for 10 min with 20 mM bromodeoxyuridine (BrdU; Amersham, Buckinghamshire, UK). Harvested cells were fixed overnight with 70% ethanol at 4°C and successively incubated as follows: (i) in 2 N HCl and 0.5% Triton X-100 for 30 min at room temperature; (ii) in FITC-conjugated anti-BrdU antibody (Pharmingen, San Diego, CA) for 1 h at room temperature; and (iii) in 5 µg/mL propidium iodide (PI) in PBS. Cells were washed with PBS containing 2% FBS and 0.1% sodium azide between each incubation. Subsequent flow-cytometric analysis was performed with a FACScan (Becton Dickinson, Mountain View, CA, USA). Fluorescence data were displayed as dot plots using Cell Quest software (Becton Dickinson).

### Measurement of Cellular Sensitivity

10^4^ cells were seeded into 24-well plates containing 1 mL per well of culture medium and incubated at 39.5°C. ATP assays were carried out with 96-well plates using a CellTiter-Glo Luminescent Cell Viability Assay Kit (Promega Corp., Madison, WI, USA) at 48 h after chemical exposure. Briefly, we transferred 100 µL cell suspensions to the individual wells of the 96-well plates, held the plates at room temperature for approximately 30 min, added 100 µL of CellTiter-Glo reagent, and mixed the contents for 2 min on an orbital shaker to induce cell lysis. The plate was then incubated at room temperature for 10 min to stabilize the luminescent signal. We measured luminescence using a Fluoroskan Ascent FL fluorometer (Thermo Fisher Scientific Inc., Waltham, MA, USA).

## Results

### DSB-repair-proficient and -deficient Cells Show Similar Sensitivity to Three Replication-blocking Agents: Aphidicolin, 5-FU, and HU

**Figure 1:**
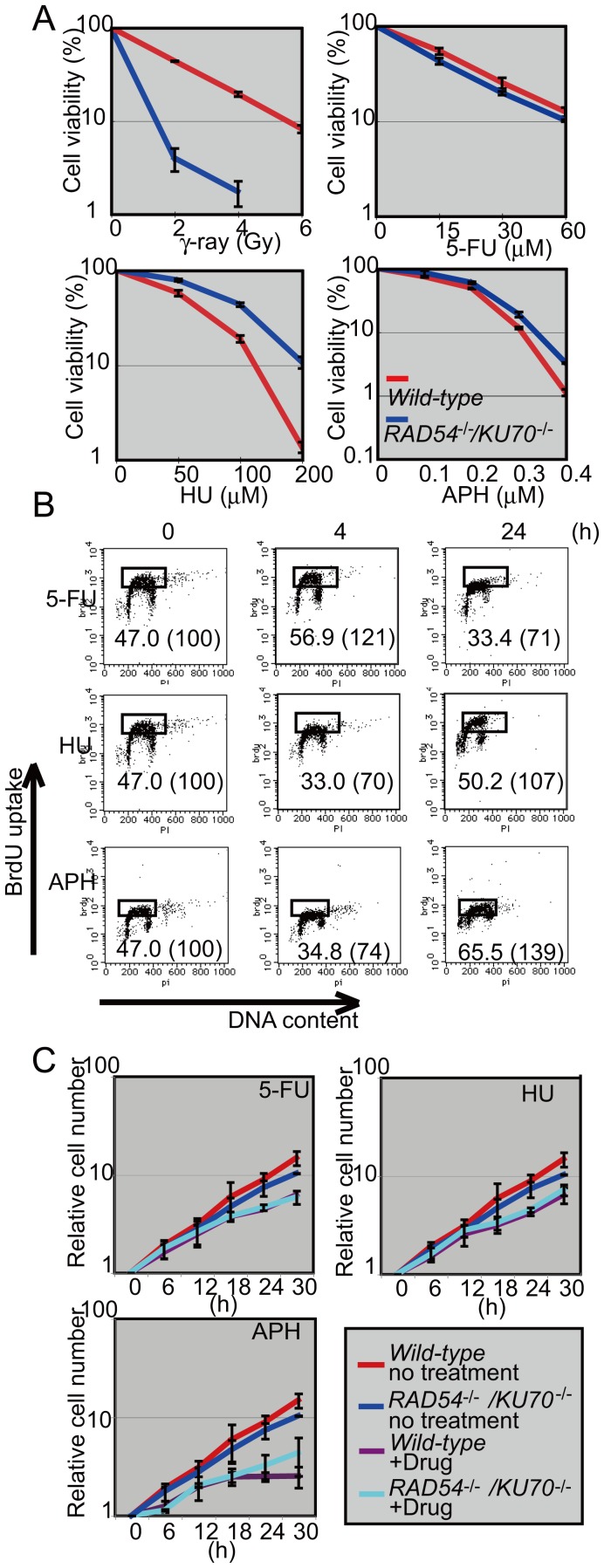
Comparable sensitivity to 5-FU, HU, and aphidicolin for *wild-type* and *RAD54^−/−/^KU70^−/−^* DT40 cells. (A) Indicated cells were either irradiated with γ-rays and cultured for 48 h or continuously incubated with aphidicolin for 72 h or, with 5-FU or HU for 48 h. Living cells were measured in terms of level of cellular ATP. The average for three independent experiments is shown. Error bars show the standard deviation for three independent experiments. (B) Cell-cycle analysis of *wild-type* DT40 cells after treatment for 4 h and 24 h with 45 µM 5-FU, 25 µM HU, and 0.25 µM aphidicolin (APH). The x-axis represents the intensity of PI staining (linear scale) and the y-axis represents bromodeoxyuridine (BrdU) uptake during 10 min pulse-labeling (logarithmic scale). The BrdU-positive fraction defined by the square was quantified. (C) Growth curves for *wild-type* DT40 and *RAD54^−/−/^KU70^−/−^* cells over 0 to 30 h exposure to 0.25 µM aphidicolin, 45 µM 5-FU, and 25 µM HU. The number of live cells was counted every 6 h. The average of three independent experiments is shown. Error bars show the standard deviation for three independent experiments.

To investigate whether or not DSBs are induced by aphidicolin, 5-FU, and HU, we measured the viability of *wild-type* DT40 cells and the *KU70^−/−^*, *RAD54^−/−^* and *KU70^−/−/^RAD54^−/−^* clones exposed to these agents. Remarkably, cytotoxicity resulting from the three agents was comparable for these clones (Figure 1A and S2). This is in marked contrast to the effects of ionizing irradiation, which killed *KU70^−/−/^RAD54^−/−^* cells to a significantly higher degree than it did *wild-type* cells (Figure 1A and S2). Given the fact that unrepaired DSBs effectively trigger apoptosis, the comparable sensitivity between DSB-repair-deficient and -proficient cells indicates that cytotoxicity caused by the three agents probably does not result from DSBs.

We next analyzed the effect of each of the three replication-blocking agents on the cell cycle. To assess DNA replication, we measured the uptake of BrdU, a thymidine analog, after 4 and 24 h exposure to the three agents (Figure 1B). Treatment with 45 µM 5-FU did not affect BrdU uptake at 4 h and partially repressed uptake at 24 h. Treatment with 25 µM HU and 0.25 µM aphidicolin partially repressed BrdU uptake at 4 h but did not affect uptake at 24 h. Treatment with aphidicolin, 5-FU, and HU delayed cellular proliferation only transiently (Figure 1C). In summary, cells exposed to the three replication-blocking agents were capable of continuously proliferating despite the strong cytotoxicity of these agents.

### Aphidicolin, 5-FU, and HU Induce Comparable Numbers of Visible Chromosomal Breaks for Both DSB-repair-proficient and -deficient Chicken DT40 Cell Lines

**Figure 2:**
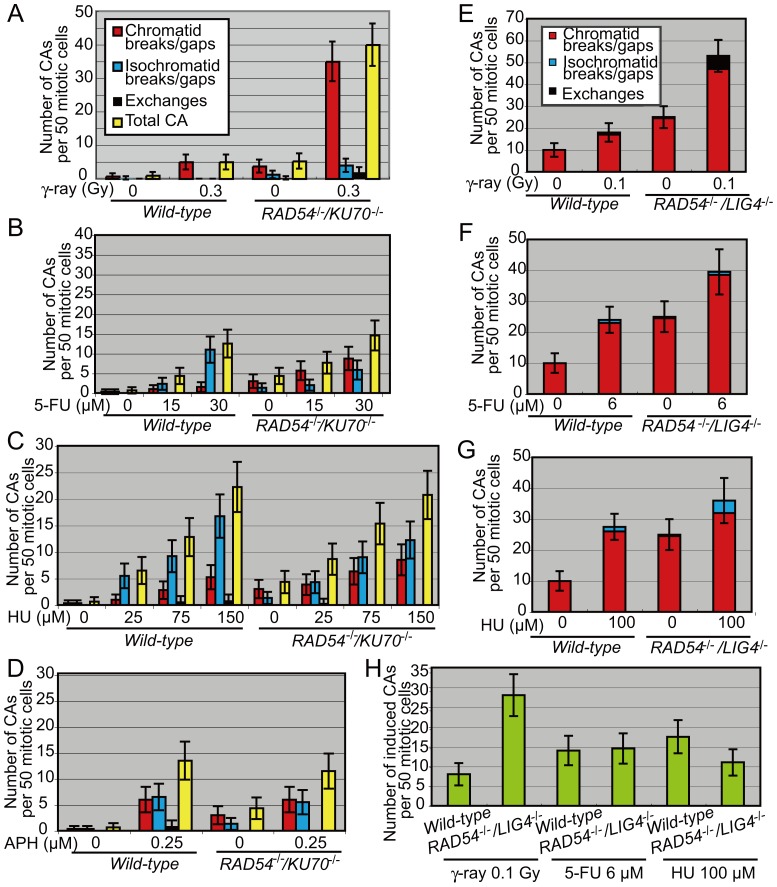
Replication-blocking agents induce comparable numbers of chromosome breaks in both DSB-repair-proficient and -deficient chicken DT40 and human Nalm-6 cell lines. (A–D) Frequency of chromosomal aberrations (CAs) in *wild-type* and *RAD54^−/−/^KU70^−/−^* DT40 cells before (0) and after treatment with (A) γ-irradiation, (B) 5-FU, (C) HU, and (D) aphidicolin (APH). Cells were analyzed at 3 h after irradiation (A). Cells were incubated with 5-FU or HU for 24 h, or with aphidicolin for 48 h at the indicated concentrations (B–D). In each case, cells were treated with colcemid for the last 3 h. More than 100 cells were analyzed in each case. (E–H) Frequency of chromosomal aberrations (CAs) in *wild-type* and *RAD54^−/−/^LIG4^−/−^* Nalm-6 cells before (0) and after treatment with (E) γ-irradiation, (F) 5-FU, and (G) HU. Cells were incubated with 5-FU or HU for 48 h at the indicated concentrations (F, G). (H) The number of induced CAs was calculated by subtracting the number of non-treated cells from the number of cells treated with γ-rays or chemicals. More than 50 cells were analyzed at 8 h after irradiation at 0.1 Gy. More than 100 cells were analyzed for 5-FU and HU. Error bars show standard error, based on the Poisson distribution of spontaneous chromosomal aberrations observed previously [37], [52].

The three replication-blocking agents all induce chromosomal breaks. To explore the cause of these induced chromosomal breaks, we measured the number of visible chromosomal breaks in *wild-type* and *RAD54^−/−/^KU70^−/−^* cells exposed to the three agents and ionizing-radiation (Figure 2A). *RAD54^−/−/^KU70^−/−^* cells exposed to 0.3 Gy ionizing radiation had eight times more chromosomal breaks than did the *wild-type* cells (Figure 2A). Thus these chromosomal breaks indeed represent unrepaired DSBs. In marked contrast, the number of visible chromosomal breaks induced by the three replication-blocking agents was comparable for *wild-type* and *RAD54^−/−/^KU70^−/−^* cells (Figure 2 B–D, Figure S1). Likewise, viability was also comparable for *wild-type* and *RAD54^−/−/^KU70^−/−^* cells (Figure S3A–D). We therefore conclude that a large percentage of chromosomal breaks produced by the three replication-blocking agents did not result from DSBs.

### 5-FU and HU Induce Similar Numbers of Chromosomal Breaks in Both DSB-repair-proficient and -deficient Human Nalm-6 Cell Lines

To confirm that the results were reproducible in human cells, we conducted chromosomal analysis using the human Nalm-6 pre-B cell line [31]. *RAD54^−/−/^LIG4^−/−^* cells consistently showed more than two times higher number of chromosomal breaks than did *wild-type* cells at 8 h after treatment with 0.1 Gy ionizing radiation (Figure 2E). We also measured the number of visible chromosomal breaks after 48 h exposure to 5-FU or HU (Figure 2F, G). By contrast, 5-FU and HU induced comparable numbers of chromosomal breaks in *wild-type* and *RAD54^−/−/^LIG4^−/−^* cells (Figure 2H). These results are consistent with those found for DT40 cells.

To further understand the occurrence of DSBs, we counted the number of subnuclear foci carrying phosphorylation of H2AX (γH2AX foci) after 48 h exposure to aphidicolin. The number of γH2AX foci found in both *wild-type* and *KU70^−/−/^RAD54^−/−^* cells was comparable (Figure S4). Thus, as with mitotic chromosomal breaks, γH2AX may represent not only DSBs but other lesions as well, which idea agrees with the fact that phosphorylation of H2AX occurs at single-stranded DNA regions that arise during replication [38]–[40]. We therefore decided not to analyze γH2AX foci in subsequent studies.

### Analysis of Chromosomal Breakage after Removal of Replication-blocking Agents

We next addressed the possibility that the inhibition of DNA replication interfered with DSB repair, since DSB repair is associated with DNA synthesis [41]. To explore this possibility, we analyzed chromosomal breaks and quantified cell viability under conditions that provide cells with additional time to repair DNA damage in aphidicolin-free medium after exposure of cells to 0.25 µM aphidicolin for 48 h (Figure S5A). It should be noted that only aphidicolin was suitable for this experiment, because elimination of aphidicolin but not of 5-FU or HU leads to immediate restoration of DNA replication. *Wild-type* cells and *RAD54^−/−/^KU70^−/−^* clones displayed comparable viabilities at both 12 and 24 h during incubation in aphidicolin-free medium, which stands in remarkable contrast to the fact that viability of the *KU70^−/−/^RAD54^−/−^* cells was three times lower than that of the *wild-type* cells after ionizing radiation (Figure S5B).

**Figure 3:**
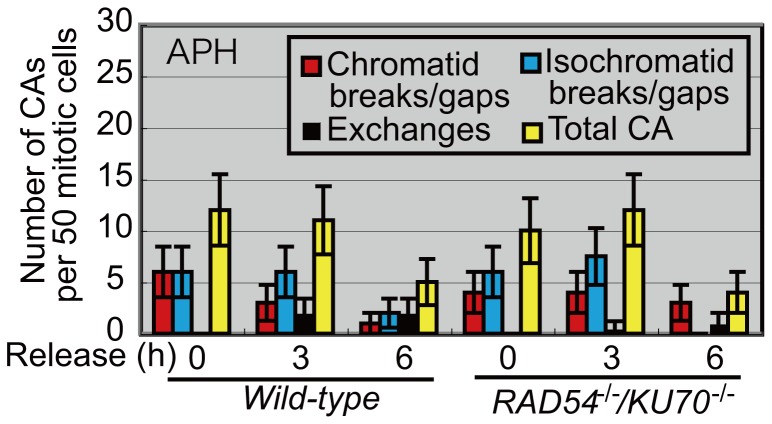
Comparable chromosomal breakage after removal of replication-blocking agents. Cells were exposed to 0.25 µM aphidicolin (APH) for 48 h. Frequency of chromosomal aberrations (CAs) for *wild-type* DT40 cells and *RAD54^−/−/^KU70^−/−^* cells is shown. More than 50 cells were analyzed in each case. Error bars show the standard error for the number of CAs in 50 mitotic cells, calculated as in Figure 2. In each case, cells were incubated with colcemid for the last 3 h.

We next measured visible chromosomal breaks at 3 and 6 h after release from the 48 h treatment with aphidicolin (Figure 3). The number of chromosomal aberrations decreased over time, with similar kinetics for both *wild-type* and *KU70^−/−/^RAD54^−/−^* clones. The data as well as the comparable viability of the two clones confirmed our conclusion that visible chromosomal breaks do not always occur as a consequence of DSBs.

### Complete Replication Blockage by Aphidicolin Followed by Release from the Blockage May Induce DSBs

**Figure 4:**
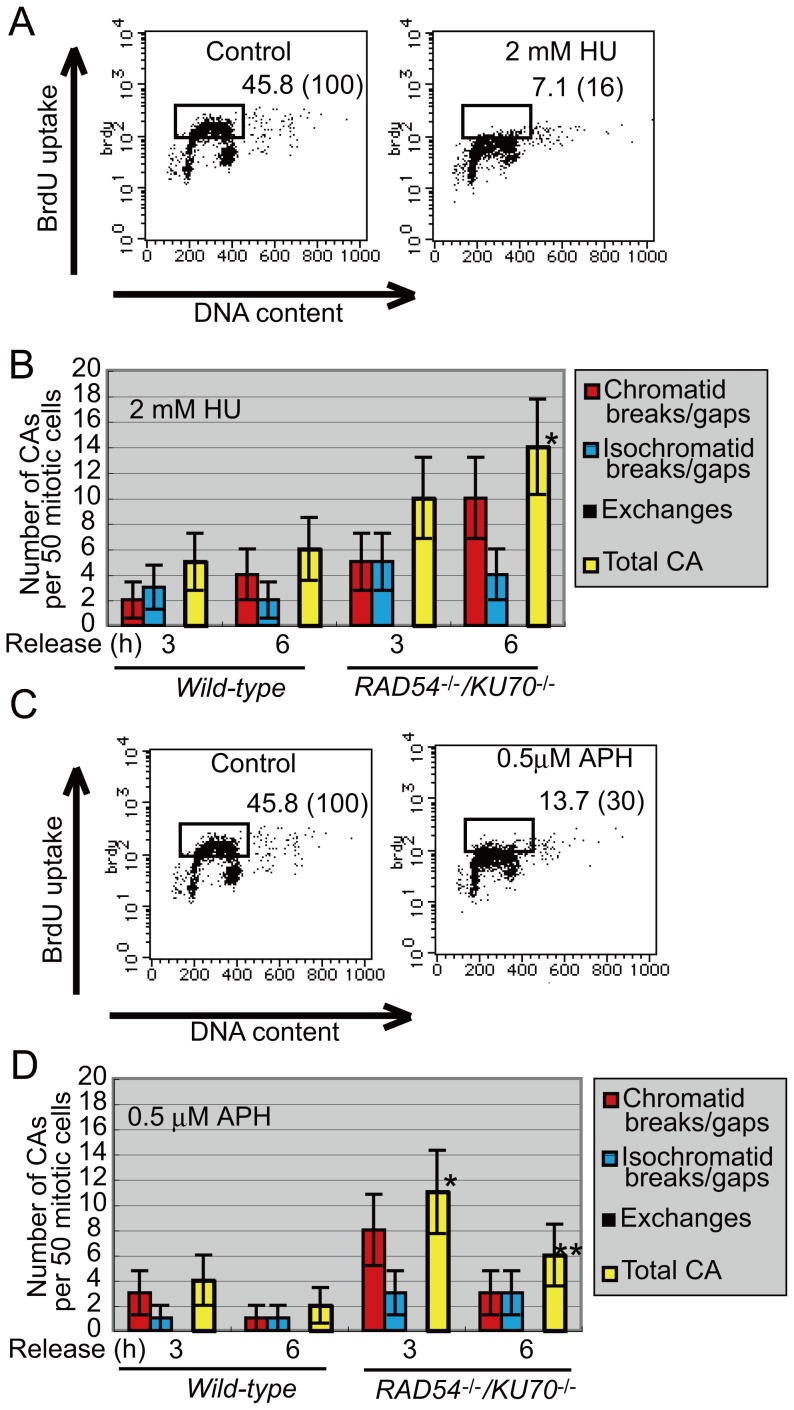
Release from complete replication blockage by a high concentration of aphidicolin or HU induces DSBs. (A) Cell-cycle analysis after treatment with 2 mM HU for 2 h. The BrdU-positive fraction was quantified as in Figure 1. (B) Frequency of chromosomal aberrations (CAs) for *wild-type* DT40 cells and *RAD54^−/−/^KU70^−/−^* cells. Cells were exposed to 2 mM HU for 2 h and then released in a drug-free medium for 3 or 6 h. (C) Cell-cycle analysis after treatment for 2 h with 0.5 µM aphidicolin (APH). The BrdU-positive fraction was quantified as in Figure 1. (D) Frequency of chromosomal aberrations (CAs) for *wild-type* DT40 cells and *RAD54^−/−/^KU70^−/−^* cells. Cells were exposed with 0.5 µM aphidicolin (APH) for 2 h and released in a drug-free medium for 3 or 6 h. More than 50 cells were analyzed in each case. Error bars show standard error for the number of CAs in 50 mitotic cells, calculated as in Figure 2. Asterisk and double asterisk: significant difference compared with *wild-type cells* (P<0.05).

Previous papers have suggested that DSBs are induced when DNA replication is completely blocked by exposing cells to a higher concentration (2 mM) of HU than the concentrations shown in Figure 2C [42], [43]. The data shown in Figure 2C indicate that the majority of visible chromosomal breaks induced by lower concentrations of HU do not result from DSBs. To investigate whether the results reported in these previous papers could be duplicated in DT40 cells, we treated *wild-type* cells and *KU70* ^−/−/^ *RAD54* ^−/−^ clones with 2 mM HU for 2 h, which treatment blocked DNA replication completely (Figure 4A). At 3 and 6 h after release from the replication blockage, we measured the number of mitotic chromosomal breaks (Figure 4B). The *KU70* ^−/−/^ *RAD54* ^−/−^ mutant exhibited more visible chromosomal breaks than did the *wild-type* cells. This result suggests that a portion of the chromatid breaks resulted from DSBs. We repeated the experiment using 0.5 µM aphidicolin instead of 2 mM HU. We blocked DNA replication by exposing cells to 0.5 µM aphidicolin (Figure 4C) for 2 h, then cultured cells with aphidicolin-free media and measured the number of mitotic chromosomal breaks at 3 and 6 h after the removal of aphidicolin (Figure 4D). Again, the *KU70* ^−/−/^ *RAD54* ^−/−^ mutant exhibited more visible chromosomal breaks than did the *wild-type* cells. Thus the release from the complete replication blockage by aphidicolin and HU may induce DSBs.

### The Inactivation of PIF1 Causes Hypersensitivity to Replication-blocking Agents

To gain insight into the molecular mechanisms underlying chromosomal breakage not resulting from DSBs, we analyzed DT40 cells deficient in PIF1 helicase or ATRIP, the essential component of the ATR kinase.

**Figure 5:**
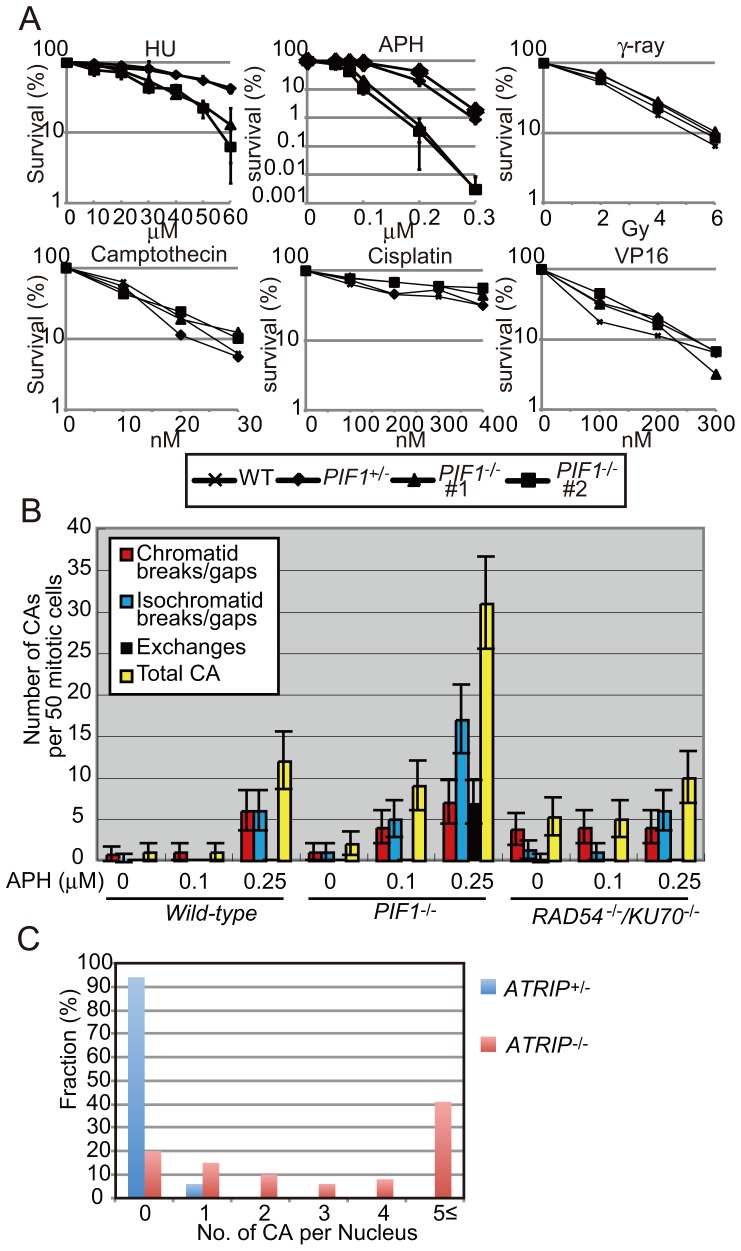
Contribution of PIF1 and ATRIP to the prevention of chromosomal breakage. (A) Cells with the indicated genotype were exposed to the indicated replication-blocking agents and DNA damage agents. The dose of the agents is displayed on the x-axis on a linear scale, while the percent fraction of surviving cells is displayed on the y-axis on a logarithmic scale. Error bars show standard deviation of mean for three independent assays. (B) Frequency of chromosomal aberrations (CAs) in *wild-type*, *PIF1^−/−^*, and *RAD54^−/−/^KU70^−/−^* DT40 cells before (0) and after treatment with aphidicolin at indicated concentration for 48 h. (C) Percentage of the cells carrying the indicated number of chromosomal breaks is indicated as a histogram. Indicated cells were treated with 0.1 µM aphidicolin (APH) for 24 h. More than 50 cells were analyzed in each case. Error bars show standard error for the number of CAs in 50 mitotic cells, calculated as in Figure 2.

We generated *PIF1^−/−^* DT40 cells by inserting a marker gene in exon9, which results in the deletion of the essential helicase domain (Figure S6A and B). The resulting *PIF1^−/−^* DT40 cells were able to proliferate with normal kinetics (Figure S6C). Remarkably, the *PIF1^−/−^* cells were hypersensitive to the replication-blocking agents (aphidicolin and HU) but not to any other DNA-damaging agents (Figure 5A). We then measured chromosomal breakage. The *PIF1^−/−^* cells exhibited significant increases in the number of chromosomal breaks after exposure to 0.1 µM aphidicolin (Figure 5B), which concentration had no effect on the cell cycle of either *wild-type* or *KU70* ^−/−/^ *RAD54* ^−/−^ cells. Thus, the chromosomal breaks observed in the *PIF1^−/−^* cells may represent unreplicated DNA sequences, since PIF1 is required for the completion of DNA replication when forks slow down [33]–[35]. A possible scenario is that the unreplicated DNA sequences might interfere with local chromosomal condensation, and thereby cause isochromatid-type breaks, cytologicaly visible discontinuity in both sister chromatids at the same location, shown in Figure 5B.

We next measured chromosomal breakage induced by aphidicolin in ATRIP-deficient cells, where the *ATRIP* gene is excised by the Cre recombinase upon addition of tamoxifen to the culture medium [44]. Taking into account the fact that *ATRIP^−/−^* cells stop proliferating at 72 h after addition of tamoxifen, prior to cell death due to genome instability, we analyzed the mitotic chromosomes at 48 h after addition of tamoxifen. We exposed the *ATRIP^−/−^* cells to aphidicolin for 24 h prior to mitotic chromosome analysis. Surprisingly, exposure to 0.1 µM aphidicolin induced extensive chromosome breakage without any detectable suppression of DNA replication (Figure 1, 5C). Over 40% of the mitotic *ATRIP^−/−^* cells showed five or more mitotic chromosomal breaks (Figure 5C). These data again supports the notion that aphidicolin-induced mitotic chromosomal breaks can result from single-strand gaps due to defective completion of DNA replication, since ATR/ATRIP prevents replication fork collapse under replication stress and thereby contributes to the re-start and completion of DNA replication.

## Discussion

We here show that the partial repression of DNA replication by aphidicolin, 5-FU, and HU induces chromosomal breaks in mitotic cells during the cell cycle. Remarkably, the partial repression induced a comparable number of chromosomal breaks in both the DSB-repair-deficient cells (human *RAD54^−/−/^LIG4^−/−^* and chicken *RAD54^−/−/^KU70^−/−^* cell lines) and their *wild-type* controls (Figure 2). This result is in marked contrast with the finding that the number of chromosomal breaks induced by γ-rays in *RAD54^−/−/^KU70^−/−^* DT40 cells was more than eight times larger than the number found in the *wild-type* control (Figure 2A). We therefore conclude that, unlike γ-ray-induced chromosomal breaks, aphidicolin, 5-FU, and HU can induce chromosomal breaks that are not subject to the major DSB repair pathways: RAD54-dependent HR and KU70- or LIG4-dependent NHEJ. This shows that there are two types of mitotic chromosomal breaks: those that result from DSBs and those that do not.

5-FU and HU are widely used for chemotherapy. Our data suggest that there are two distinct mechanisms underlying the cytotoxic effects of these agents. First, high concentrations of these agents stall replication, leading to replication collapse and DSB formation. Indeed, treatment with 2 mM HU for 2 h followed by incubation of the cells in drug-free media induced a higher number of mitotic chromosomal breaks in *KU70^−/−/^RAD54^−/−^* cells than in *wild-type* cells (Figure 4B), indicating that a portion of the induced chromosomal breaks may result from DSBs. A recent report showed that prolonged treatment (∼24 h) with a high concentration of HU (2 mM) resulted in replication fork collapse and formation of DSBs that were repaired by HR [42]. Second, treatment with lower concentrations of HU, in which replication fork progression was slowed but not completely inhibited (Figure 1B), induced mitotic chromosomal breaks that were not associated with DSBs (Figure 2). In summary, replication stress induces two different types of mitotic chromosomal breaks, depending on the concentration of the replication-blocking agent. It should be noted that the concentrations of HU employed for the experiments illustrated by Figure 1C were similar to the serum concentrations of HU used for chemotherapeutic treatment (100–300 µM) [45]. Thus, the chemotherapeutic effects of 5-FU and HU may not result from DSB formation, even though chemotherapy by these agents efficiently induces chromosomal breaks in mitotic cells. An unanswered question is, how do these agents have therapeutic effects on malignant cells when they neither stop DNA replication nor induce DSBs? One possible scenario is that chronic replication stress induces senescence manifested by cell-cycle arrest [46], collisions between replication forks and transcription [47], or mis-segregation of sister chromatids during mitosis [48].

Another pressing question is, what is the molecular mechanism for the generation of chromosomal breaks without associating DSBs? One possible answer is that even when bulk chromosomal replication is not compromised (Figure 1B), replication might not be completed at regions with low origin-density and replication barriers such as DNA sequences prone to secondary structure formation, which correspond to common fragile chromosome sites [49], [50]. The resulting unreplicated single-strand DNA gaps might interfere with local chromosome condensation and thereby induce cytogenetically visible break sites. This scenario is supported by the fact that PIF1, which facilitates replication-fork progression, repressed the formation of mitotic chromosomal breakage (Figure 5B). Moreover, a previous study showed that premature condensation of chromosomes indeed induces chromosomal breaks in mitotic cells [51]. In summary, we posit that replication stress caused by aphidicolin and therapeutic concentrations of 5-FU and HU can induce chromosomal breakage that is not associated with DSBs. The molecular mechanism for the cytotoxicity of these chemotherapeutic agents is a topic for future experimentation.

## Supporting Information

Figure S1 **Representative image of the cytogenetically visible chromosome breaks.** *Wild-type* DT40 cells were treated with HU as in Figure 2. Representative chromatid-type break and isochromatid-type break was indicated by arrowhead and arrow respectively.
(TIF)

Figure S2 **Comparable sensitivity to 5-FU, HU, and aphidicolin for** ***wild-type*** **,** ***RAD54^−/−^*** **,** ***KU70^−/−^*** **and** ***RAD54^−/−/^KU70^−/−^*** **DT40 cells.** Indicated cells were either irradiated with g-rays and cultured for 48 h or continuously incubated with aphidicolin for 72 h or, with 5-FU or HU for 48 h. Living cells were measured in terms of level of cellular ATP. The average for three independent experiments is shown. Error bars show the standard deviation for three independent experiments.
(TIF)

Figure S3 **Quantitative analysis of cell viability after 24 h treatment with aphidicolin, 5-FU, and HU.** (A) Dot plots represent the intensity of Annexin V fluorescent staining on the x axis (logarithmic scale) and the intensity of propidium-iodine (PI) staining on the y axis (logarithmic scale). (B–D) Numbers indicate the percentages of live, preapoptotic, and dead cells defined by Annexin V−/PI-, Annexin V+/PI-, and PI+ staining, respectively after (B) 5-FU, (C) HU, and (D) aphidicolin (APH) treatment. The average for three separate experiments is shown. Error bars show the standard deviation for three independent experiments.
(TIF)

Figure S4 **Comparable number of gH2AX foci following replication stress.** Percentage of cells carrying the indicated number of γH2AX foci is shown as histogram. Indicated cells were treated with aphidicolin (APH) for 48 h.
(TIF)

Figure S5 **Cell viability after removal of replication-blocking agents.** (A, B) Cells were exposed to 0.25 µM aphidicolin (APH) for 24 h (A) or were irradiated with 2 Gy of γ-ray (B) and released in a drug-free medium for 12 or 24 h. Numbers indicate the percentages of live, preapoptotic, and dead cells, as in Fig. S1.
(TIF)

Figure S6 ***PIF1*** **disruption in DT40 cells.** (A) A *neo* or *bsr* selection-marker gene was inserted in the *wild-type* chicken *PIF1* locus exon 7. The targeting construct is shown and compared with the relevant chicken *PIF1* genomic sequences (top). Open boxes indicate the position of the exons. Relevant *Stu*I sites and the position of the probe used in the Southern blot analysis are indicated. (B) Disruption of *PIF1* was confirmed by Southern blot. (C) Relative growth rate plotted for the indicated genotypes. Error bars show the standard deviation of mean for three independent experiments.
(TIF)

## References

[1] Cimino MC (2006) Comparative overview of current international strategies and guidelines for genetic toxicology testing for regulatory purposes. Environ Mol Mutagen 47: 362–390. 16649190 10.1002/em.20216

[2] Trujillo JM, Ahearn MJ, Cork A (1974) General implications of chromosomal alterations in human leukemia. Hum Pathol 5: 675–686. 4608960 10.1016/s0046-8177(74)80038-9

[3] Sachs RK, Hlatky LR, Trask BJ (2000) Radiation-produced chromosome aberrations: colourful clues. Trends Genet 16: 143–146. 10729825 10.1016/s0168-9525(99)01960-5

[4] ISCN (1985) Report of the standing committee on human cytogenetic nomenclature. Birth Defects Orig Artic Ser 21: 1–117. 4041569

[5] Yamamoto KN, Hirota K, Kono K, Takeda S, Sakamuru S, et al (2011) Characterization of environmental chemicals with potential for DNA damage using isogenic DNA repair-deficient chicken DT40 cell lines. Environ Mol Mutagen 52: 547–561. 21538559 10.1002/em.20656 PMC3278799

[6] Gebhart E (1981) Sister chromatid exchange (SCE) and structural chromosome aberration in mutagenicity testing. Hum Genet 58: 235–254. 6459983 10.1007/BF00294917

[7] Takata M, Sasaki MS, Sonoda E, Morrison C, Hashimoto M, et al (1998) Homologous recombination and non-homologous end-joining pathways of DNA double-strand break repair have overlapping roles in the maintenance of chromosomal integrity in vertebrate cells. EMBO J 17: 5497–5508. 9736627 10.1093/emboj/17.18.5497 PMC1170875

[8] Deckbar D, Birraux J, Krempler A, Tchouandong L, Beucher A, et al (2007) Chromosome breakage after G2 checkpoint release. J Cell Biol 176: 749–755. 17353355 10.1083/jcb.200612047 PMC2064048

[9] Bender MA, Griggs HG, Bedford JS (1974) Mechanisms of chromosomal aberration production. III. Chemicals and ionizing radiation. Mutat Res 23: 197–212. 4275776 10.1016/0027-5107(74)90140-7

[10] Bryant PE, Johnston PJ (1993) Restriction-endonuclease-induced DNA double-strand breaks and chromosomal aberrations in mammalian cells. Mutat Res 299: 289–296. 7683096 10.1016/0165-1218(93)90105-m

[11] Iliakis G, Wang H, Perrault AR, Boecker W, Rosidi B, et al (2004) Mechanisms of DNA double strand break repair and chromosome aberration formation. Cytogenet Genome Res 104: 14–20. 15162010 10.1159/000077461

[12] Natarajan AT, Obe G, van Zeeland AA, Palitti F, Meijers M, et al (1980) Molecular mechanisms involved in the production of chromosomal aberrations. II. Utilization of Neurospora endonuclease for the study of aberration production by X-rays in G1 and G2 stages of the cell cycle. Mutat Res 69: 293–305. 6244487 10.1016/0027-5107(80)90094-9

[13] Richardson C, Jasin M (2000) Frequent chromosomal translocations induced by DNA double-strand breaks. Nature 405: 697–700. 10864328 10.1038/35015097

[14] Cohen MM, Shaham M, Dagan J, Shmueli E, Kohn G (1975) Cytogenetic investigations in families with ataxia-telangiectasia. Cytogenet Cell Genet 15: 338–356. 1222588 10.1159/000130530

[15] Klein IA, Resch W, Jankovic M, Oliveira T, Yamane A, et al (2011) Translocation-capture sequencing reveals the extent and nature of chromosomal rearrangements in B lymphocytes. Cell 147: 95–106. 21962510 10.1016/j.cell.2011.07.048 PMC3190307

[16] van Gent DC, Hoeijmakers JH, Kanaar R (2001) Chromosomal stability and the DNA double-stranded break connection. Nat Rev Genet 2: 196–206. 11256071 10.1038/35056049

[17] Wang JH, Gostissa M, Yan CT, Goff P, Hickernell T, et al (2009) Mechanisms promoting translocations in editing and switching peripheral B cells. Nature 460: 231–236. 19587764 10.1038/nature08159 PMC2907259

[18] Galloway SM (1994) Chromosome aberrations induced in vitro: mechanisms, delayed expression, and intriguing questions. Environ Mol Mutagen 23 Suppl 24 44–53. 8162908 10.1002/em.2850230612

[19] Ikegami S, Taguchi T, Ohashi M, Oguro M, Nagano H, et al (1978) Aphidicolin prevents mitotic cell division by interfering with the activity of DNA polymerase-alpha. Nature 275: 458–460. 692726 10.1038/275458a0

[20] Wang TSF, Conger KL, Copeland WC, Arroyo MP (1999) Eukaryotic DNA replication : a practical approach, ed Cotterill S.; Cotterill S, editor. England: Oxford; New York : Oxford University Press. 67–92 p.

[21] Amstutz U, Froehlich TK, Largiader CR (2011) Dihydropyrimidine dehydrogenase gene as a major predictor of severe 5-fluorouracil toxicity. Pharmacogenomics 12: 1321–1336. 21919607 10.2217/pgs.11.72

[22] Koc A, Wheeler LJ, Mathews CK, Merrill GF (2004) Hydroxyurea arrests DNA replication by a mechanism that preserves basal dNTP pools. J Biol Chem 279: 223–230. 14573610 10.1074/jbc.M303952200

[23] Sonoda E, Hochegger H, Saberi A, Taniguchi Y, Takeda S (2006) Differential usage of non-homologous end-joining and homologous recombination in double strand break repair. DNA Repair (Amst) 5: 1021–1029. 16807135 10.1016/j.dnarep.2006.05.022

[24] Weinstock DM, Richardson CA, Elliott B, Jasin M (2006) Modeling oncogenic translocations: distinct roles for double-strand break repair pathways in translocation formation in mammalian cells. DNA Repair (Amst) 5: 1065–1074. 16815104 10.1016/j.dnarep.2006.05.028

[25] Eppink B, Tafel AA, Hanada K, van Drunen E, Hickson ID, et al (2011) The response of mammalian cells to UV-light reveals Rad54-dependent and independent pathways of homologous recombination. DNA Repair (Amst) 10: 1095–1105. 21885354 10.1016/j.dnarep.2011.08.006

[26] Kanaar R, Troelstra C, Swagemakers SM, Essers J, Smit B, et al (1996) Human and mouse homologs of the *Saccharomyces cerevisiae RAD54* DNA repair gene: evidence for functional conservation. Curr Biol 6: 828–838. 8805304 10.1016/s0960-9822(02)00606-1

[27] Lieber MR (2010) The mechanism of double-strand DNA break repair by the nonhomologous DNA end-joining pathway. Annu Rev Biochem 79: 181–211. 20192759 10.1146/annurev.biochem.052308.093131 PMC3079308

[28] Couedel C, Mills KD, Barchi M, Shen L, Olshen A, et al (2004) Collaboration of homologous recombination and nonhomologous end-joining factors for the survival and integrity of mice and cells. Genes Dev 18: 1293–1304. 15175261 10.1101/gad.1209204 PMC420355

[29] Mills KD, Ferguson DO, Essers J, Eckersdorff M, Kanaar R, et al (2004) Rad54 and DNA Ligase IV cooperate to maintain mammalian chromatid stability. Genes Dev 18: 1283–1292. 15175260 10.1101/gad.1204304 PMC420354

[30] Evans TJ, Yamamoto KN, Hirota K, Takeda S (2010) Mutant cells defective in DNA repair pathways provide a sensitive high-throughput assay for genotoxicity. DNA Repair (Amst) 9: 1292–1298. 21030320 10.1016/j.dnarep.2010.09.017

[31] Adachi N, Kurosawa A, Koyama H (2008) Highly proficient gene targeting by homologous recombination in the human pre-B cell line Nalm-6. Methods Mol Biol 435: 17–29. 18370065 10.1007/978-1-59745-232-8_2

[32] Buerstedde JM, Takeda S (1991) Increased ratio of targeted to random integration after transfection of chicken B cell lines. Cell 67: 179–188. 1913816 10.1016/0092-8674(91)90581-i

[33] Shimada K, Gasser SM (2011) DNA replication: Pif1 pulls the plug on stalled replication forks. Curr Biol 22: R404–405. 10.1016/j.cub.2012.04.015 22625857

[34] Anand RP, Shah KA, Niu H, Sung P, Mirkin SM, et al (2012) Overcoming natural replication barriers: differential helicase requirements. Nucleic Acids Res 40: 1091–1105. 21984413 10.1093/nar/gkr836 PMC3273818

[35] Pinter SF, Aubert SD, Zakian VA (2008) The Schizosaccharomyces pombe Pfh1p DNA helicase is essential for the maintenance of nuclear and mitochondrial DNA. Mol Cell Biol 28: 6594–6608. 18725402 10.1128/MCB.00191-08 PMC2573227

[36] Wlodarska I, Aventin A, Ingles-Esteve J, Falzetti D, Criel A, et al (1997) A new subtype of pre-B acute lymphoblastic leukemia with t(5;12)(q31q33;p12), molecularly and cytogenetically distinct from t(5;12) in chronic myelomonocytic leukemia. Blood 89: 1716–1722. 9057655

[37] Sonoda E, Sasaki MS, Buerstedde JM, Bezzubova O, Shinohara A, et al (1998) Rad51-deficient vertebrate cells accumulate chromosomal breaks prior to cell death. EMBO J 17: 598–608. 9430650 10.1093/emboj/17.2.598 PMC1170409

[38] Revet I, Feeney L, Bruguera S, Wilson W, Dong TK, et al (2011) Functional relevance of the histone gammaH2Ax in the response to DNA damaging agents. Proc Natl Acad Sci U S A 108: 8663–8667. 21555580 10.1073/pnas.1105866108 PMC3102364

[39] Lobrich M, Shibata A, Beucher A, Fisher A, Ensminger M, et al (2010) gammaH2AX foci analysis for monitoring DNA double-strand break repair: strengths, limitations and optimization. Cell Cycle 9: 662–669. 20139725 10.4161/cc.9.4.10764

[40] Schwartz M, Zlotorynski E, Goldberg M, Ozeri E, Rahat A, et al (2005) Homologous recombination and nonhomologous end-joining repair pathways regulate fragile site stability. Genes Dev 19: 2715–2726. 16291645 10.1101/gad.340905 PMC1283964

[41] Kawamoto T, Araki K, Sonoda E, Yamashita YM, Harada K, et al (2005) Dual roles for DNA polymerase eta in homologous DNA recombination and translesion DNA synthesis. Mol Cell 20: 793–799. 16337602 10.1016/j.molcel.2005.10.016

[42] Petermann E, Orta ML, Issaeva N, Schultz N, Helleday T (2010) Hydroxyurea-stalled replication forks become progressively inactivated and require two different RAD51-mediated pathways for restart and repair. Mol Cell 37: 492–502. 20188668 10.1016/j.molcel.2010.01.021 PMC2958316

[43] Schlacher K, Christ N, Siaud N, Egashira A, Wu H, et al (2011) Double-strand break repair-independent role for BRCA2 in blocking stalled replication fork degradation by MRE11. Cell 145: 529–542. 21565612 10.1016/j.cell.2011.03.041 PMC3261725

[44] Shigechi T, Tomida J, Sato K, Kobayashi M, Eykelenboom JK, et al (2012) ATR-ATRIP kinase complex triggers activation of the Fanconi anemia DNA repair pathway. Cancer Res 72: 1149–1156. 22258451 10.1158/0008-5472.CAN-11-2904

[45] Beckloff GL, Lerner HJ, Frost D, Russo-Alesi FM, Gitomer S (1965) Hydroxyurea (NSC-32065) in biologic fluids: dose-concentration relationship. Cancer Chemother Rep 48: 57–58. 5834739

[46] Marusyk A, Wheeler LJ, Mathews CK, DeGregori J (2007) p53 mediates senescence-like arrest induced by chronic replicational stress. Mol Cell Biol 27: 5336–5351. 17515610 10.1128/MCB.01316-06 PMC1952086

[47] Helmrich A, Ballarino M, Tora L (2011) Collisions between replication and transcription complexes cause common fragile site instability at the longest human genes. Mol Cell 44: 966–977. 22195969 10.1016/j.molcel.2011.10.013

[48] Kawabata T, Luebben SW, Yamaguchi S, Ilves I, Matise I, et al (2011) Stalled fork rescue via dormant replication origins in unchallenged S phase promotes proper chromosome segregation and tumor suppression. Mol Cell 41: 543–553. 21362550 10.1016/j.molcel.2011.02.006 PMC3062258

[49] Freudenreich CH (2007) Chromosome fragility: molecular mechanisms and cellular consequences. Front Biosci 12: 4911–4924. 17569619 10.2741/2437

[50] Letessier A, Millot GA, Koundrioukoff S, Lachages AM, Vogt N, et al (2011) Cell-type-specific replication initiation programs set fragility of the FRA3B fragile site. Nature 470: 120–123. 21258320 10.1038/nature09745

[51] El Achkar E, Gerbault-Seureau M, Muleris M, Dutrillaux B, Debatisse M (2005) Premature condensation induces breaks at the interface of early and late replicating chromosome bands bearing common fragile sites. Proc Natl Acad Sci U S A 102: 18069–18074. 16330769 10.1073/pnas.0506497102 PMC1312387

[52] Kikuchi K, Abdel-Aziz HI, Taniguchi Y, Yamazoe M, Takeda S, et al (2009) Bloom DNA helicase facilitates homologous recombination between diverged homologous sequences. J Biol Chem 284: 26360–26367. 19661064 10.1074/jbc.M109.029348 PMC2785323

