# Supplementary figures and images for: Interference in DNA Replication Can Cause Mitotic Chromosomal Breakage Unassociated with Double-Strand Breaks
Source: PLoS One. 2013 Apr 3;8(4):e60043. doi: 10.1371/journal.pone.0060043 (PMC3616066; doi:10.1371/journal.pone.0060043)

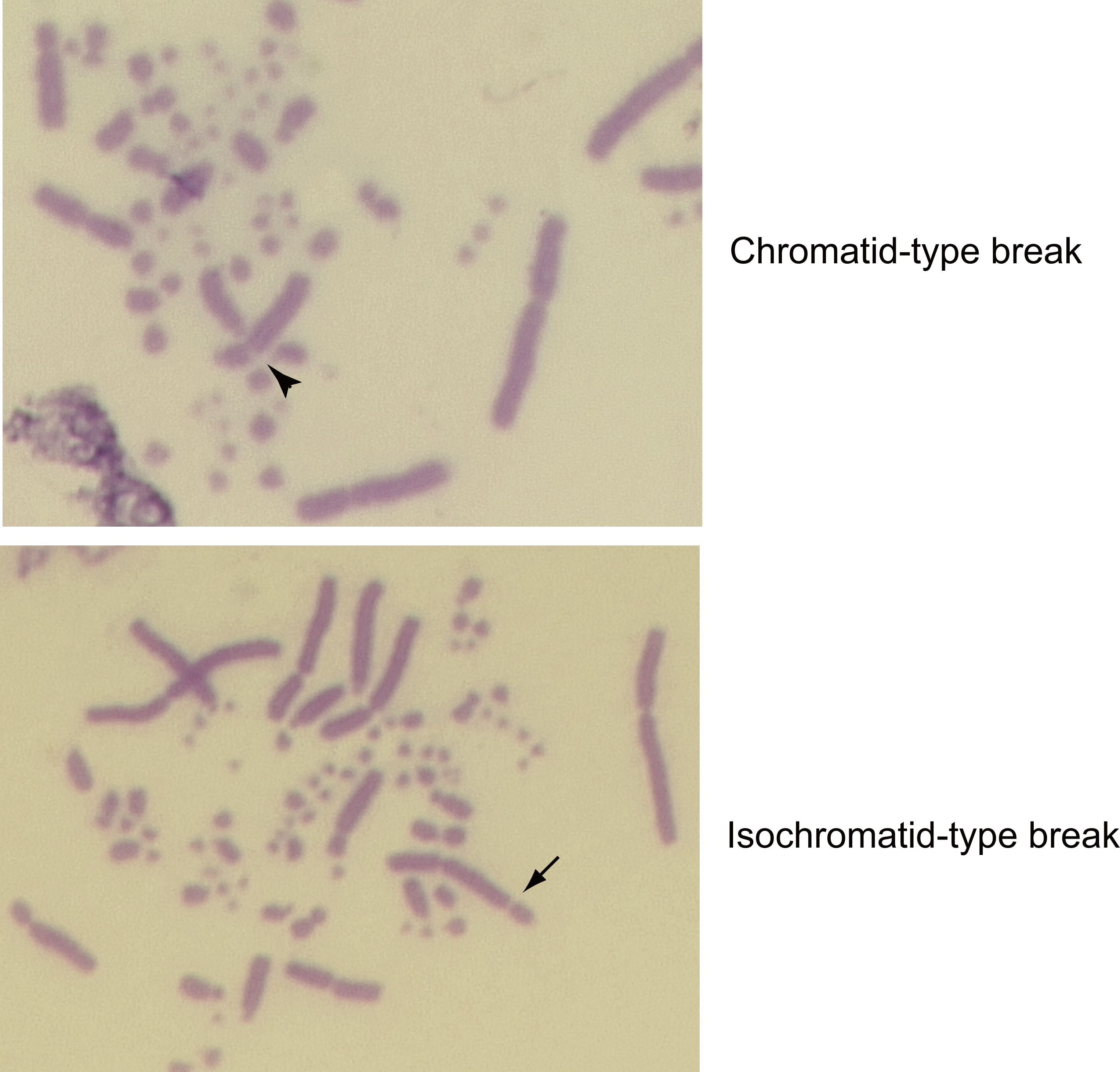

Supplement: Figure S1 — Representative image of the cytogenetically visible chromosome breaks. Wild-type DT40 cells were treated with HU as in Figure 2. Representative chromatid-type break and isochromatid-type break was indicated by arrowhead and arrow respectively. (TIF) [file pone.0060043.s001.tif]

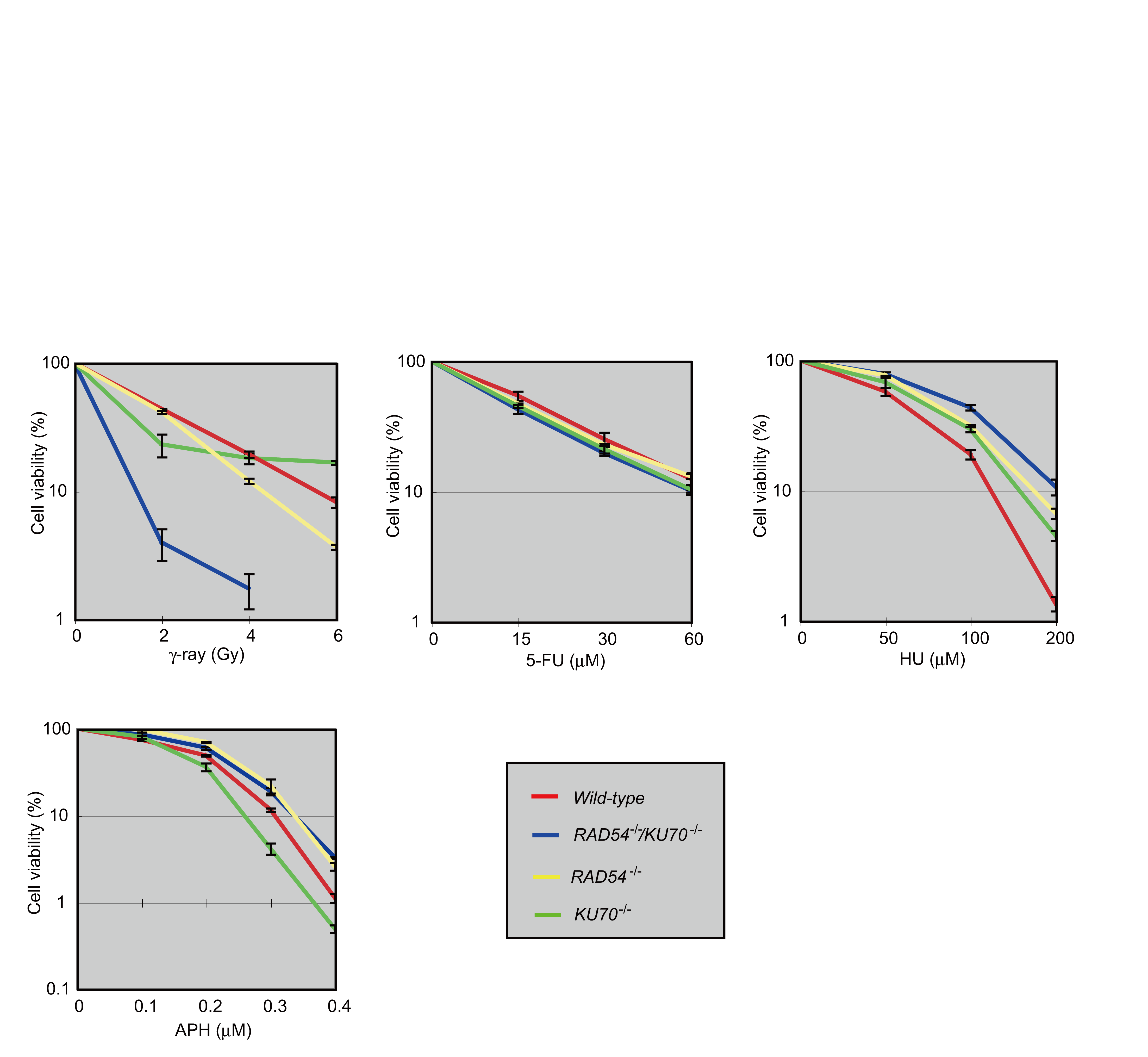

Supplement: Figure S2 — Comparable sensitivity to 5-FU, HU, and aphidicolin for wild-type , RAD54−/− , KU70−/− and RAD54−/−/KU70−/− DT40 cells. Indicated cells were either irradiated with g-rays and cultured for 48 h or continuously incubated with aphidicolin for 72 h or, with 5-FU or HU for 48 h. Living cells were measured in terms of level of cellular ATP. The average for three independent experiments is shown. Error bars show the standard deviation for three independent experiments. (TIF) [file pone.0060043.s002.tif]

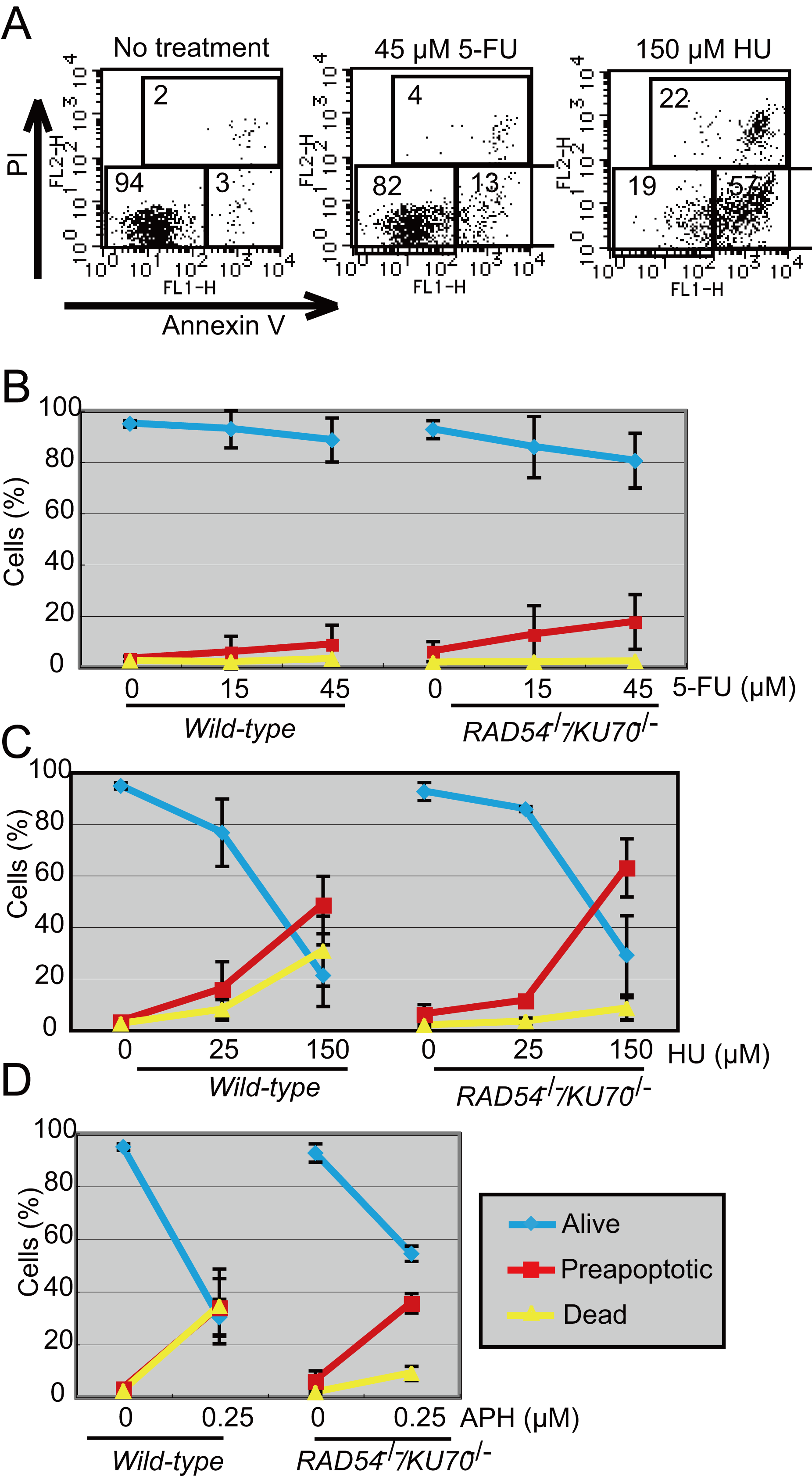

Supplement: Figure S3 — Quantitative analysis of cell viability after 24 h treatment with aphidicolin, 5-FU, and HU. (A) Dot plots represent the intensity of Annexin V fluorescent staining on the x axis (logarithmic scale) and the intensity of propidium-iodine (PI) staining on the y axis (logarithmic scale). (B–D) Numbers indicate the percentages of live, preapoptotic, and dead cells defined by Annexin V−/PI-, Annexin V+/PI-, and PI+ staining, respectively after (B) 5-FU, (C) HU, and (D) aphidicolin (APH) treatment. The average for three separate experiments is shown. Error bars show the standard deviation for three independent experiments. (TIF) [file pone.0060043.s003.tif]

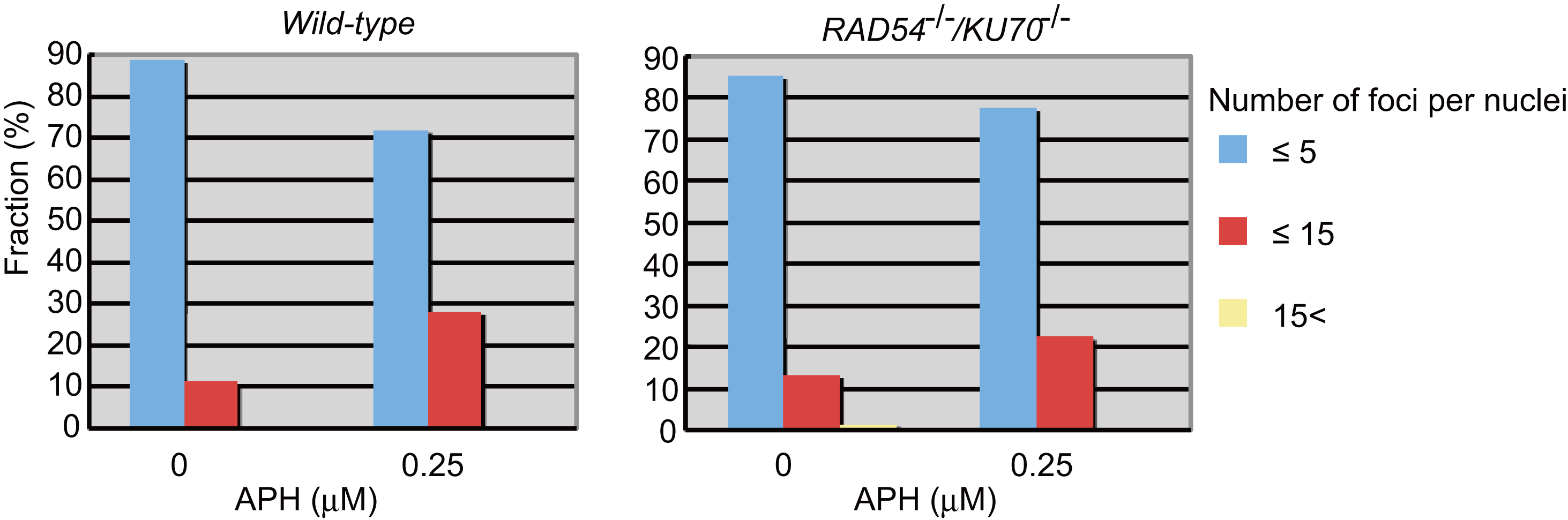

Supplement: Figure S4 — Comparable number of gH2AX foci following replication stress. Percentage of cells carrying the indicated number of γH2AX foci is shown as histogram. Indicated cells were treated with aphidicolin (APH) for 48 h. (TIF) [file pone.0060043.s004.tif]

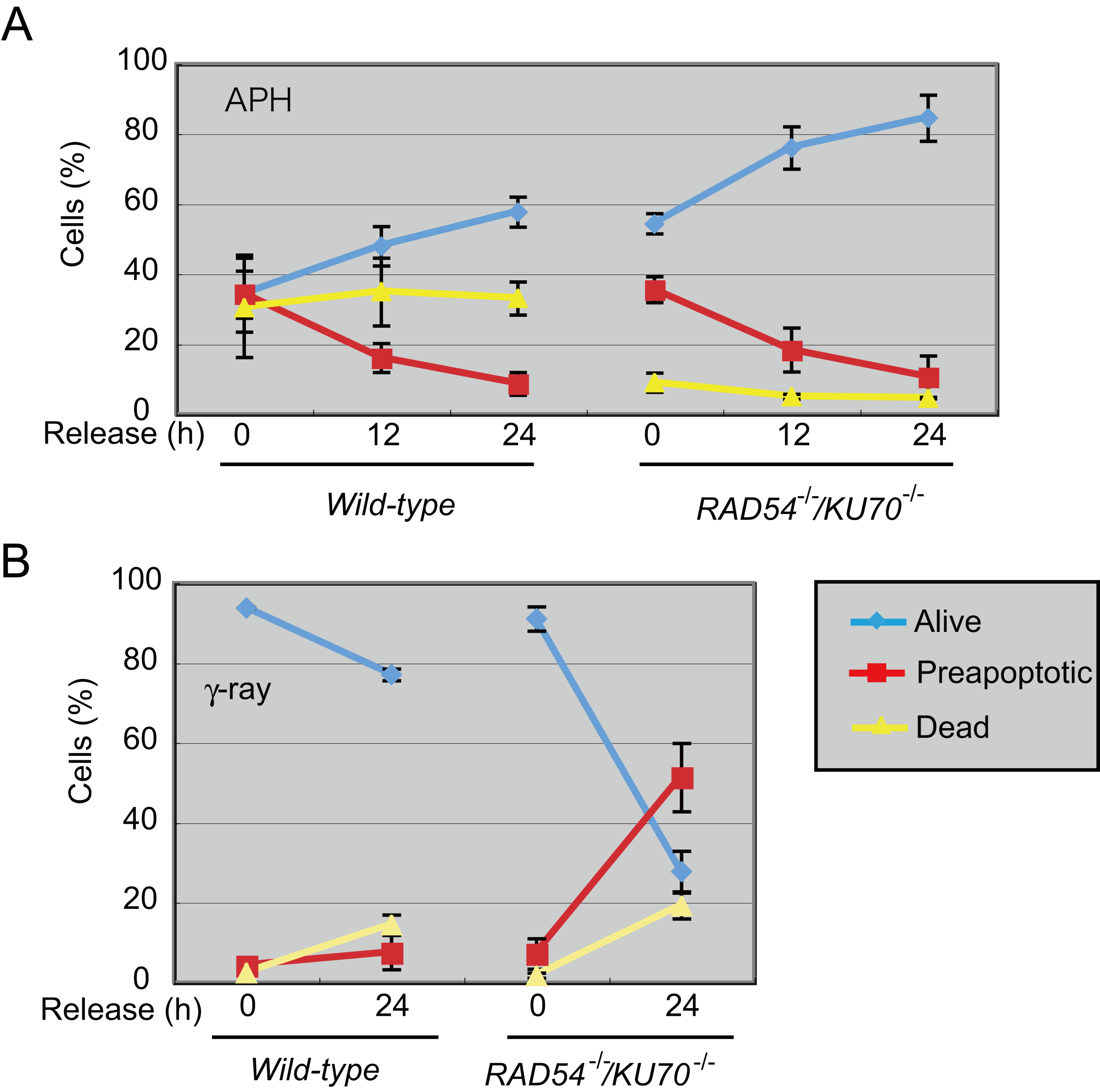

Supplement: Figure S5 — Cell viability after removal of replication-blocking agents. (A, B) Cells were exposed to 0.25 µM aphidicolin (APH) for 24 h (A) or were irradiated with 2 Gy of γ-ray (B) and released in a drug-free medium for 12 or 24 h. Numbers indicate the percentages of live, preapoptotic, and dead cells, as in Fig. S1. (TIF) [file pone.0060043.s005.tif]

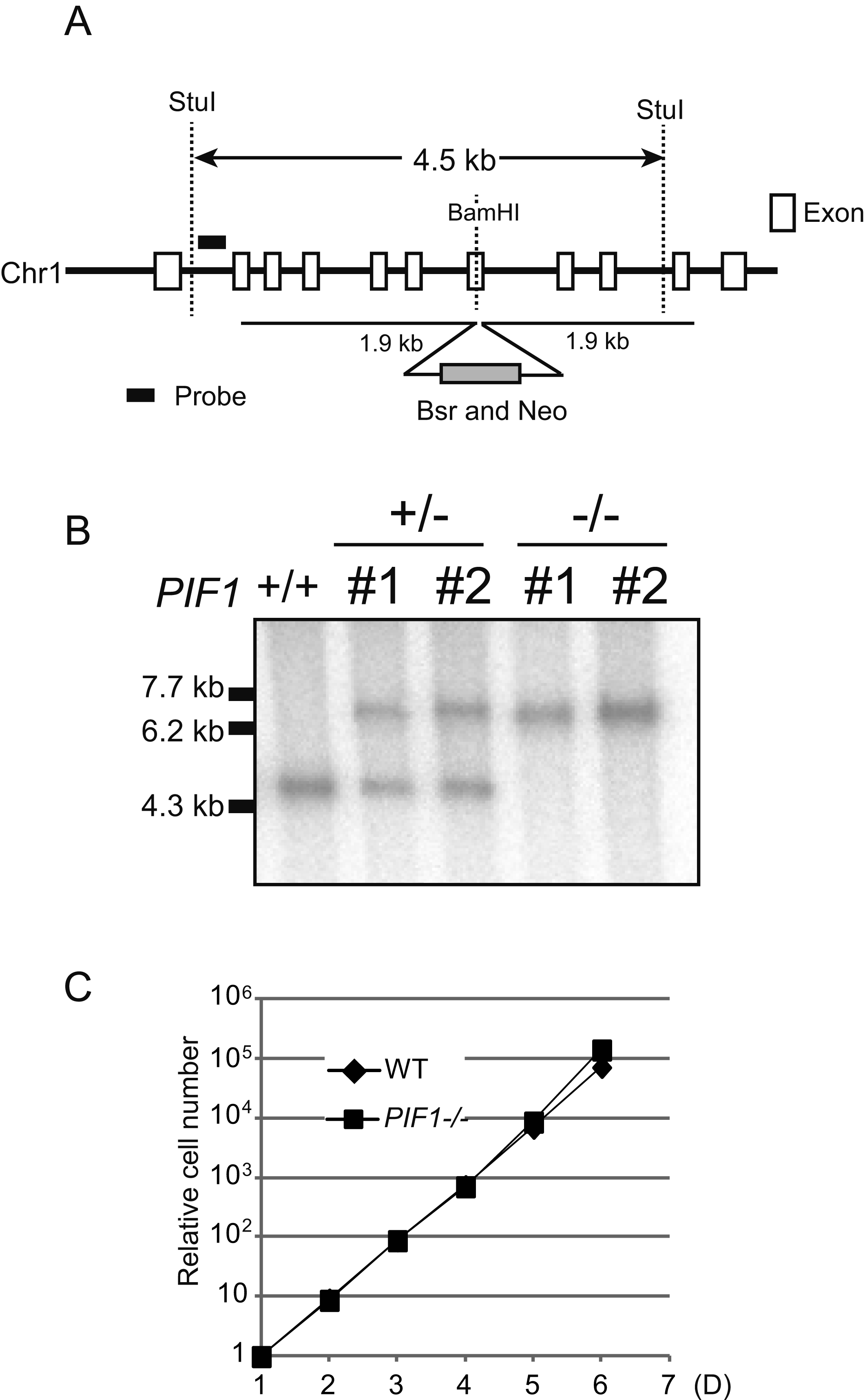

Supplement: Figure S6 — PIF1 disruption in DT40 cells. (A) A neo or bsr selection-marker gene was inserted in the wild-type chicken PIF1 locus exon 7. The targeting construct is shown and compared with the relevant chicken PIF1 genomic sequences (top). Open boxes indicate the position of the exons. Relevant StuI sites and the position of the probe used in the Southern blot analysis are indicated. (B) Disruption of PIF1 was confirmed by Southern blot. (C) Relative growth rate plotted for the indicated genotypes. Error bars show the standard deviation of mean for three independent experiments. (TIF) [file pone.0060043.s006.tif]
